# The Environment, Farm Animals and Foods as Sources of *Clostridioides difficile* Infection in Humans

**DOI:** 10.3390/foods12051094

**Published:** 2023-03-04

**Authors:** Declan Bolton, Pilar Marcos

**Affiliations:** Teagasc Food Research Centre, Ashtown, D15 DY05 Dublin, Ireland

**Keywords:** *Clostridioides difficile*, ribotypes, environment, food, epidemiology

## Abstract

The recent discovery of the same *Clostridioides difficile* ribotypes associated with human infection in a broad range of environments, animals and foods, coupled with an ever-increasing rate of community-acquired infections, suggests this pathogen may be foodborne. The objective of this review was to examine the evidence supporting this hypothesis. A review of the literature found that forty-three different ribotypes, including six hypervirulent strains, have been detected in meat and vegetable food products, all of which carry the genes encoding pathogenesis. Of these, nine ribotypes (002, 003, 012, 014, 027, 029, 070, 078 and 126) have been isolated from patients with confirmed community-associated *C. difficile* infection (CDI). A meta-analysis of this data suggested there is a higher risk of exposure to all ribotypes when consuming shellfish or pork, with the latter being the main foodborne route for ribotypes 027 and 078, the hypervirulent strains that cause most human illnesses. Managing the risk of foodborne CDI is difficult as there are multiple routes of transmission from the farming and processing environment to humans. Moreover, the endospores are resistant to most physical and chemical treatments. The most effective current strategy is, therefore, to limit the use of broad-spectrum antibiotics while advising potentially vulnerable patients to avoid high-risk foods such as shellfish and pork.

## 1. Introduction

*Clostridioides difficile* is a Gram-positive, endospore-forming anaerobic bacterium often carried asymptomatically in the human gastrointestinal tract [1,2,3,4,5]. However, when conditions are favourable, the endospores germinate in the colon, vegetative cells multiply, and toxins are produced [6], resulting in watery, non-bloody diarrhoea with abdominal pain, toxic megacolon and/or pseudomembranous colitis, which may be fatal [7,8,9].

The most common risk factor associated with CDI is the use/misuse of broad-spectrum antibiotics. *C. difficile* is often resistant to a wide range of antibiotics [10], and the administration of antibiotics like clindamycin, cephalosporins, penicillins and fluoroquinolones eliminate competitive bacteria in the colon and promote *C. difficile* outgrowth [11]. The elderly, infants, other immune compromised, and patients on antibiotic therapies are therefore most at risk [1,2,4], although the incidence of CDI in pregnant women, children and patients with inflammatory bowel disease (IBD) has also increased [12].

The generally accepted route for human CDI is transmission from the healthcare environment [13]. However, in recent years the proportion of community-acquired CDI, where the patient has no association with a healthcare facility, has increased [14]. At the same time, non-human reservoirs, including the natural environment (soil, rivers and lakes) [15] and animals, including domestic pets [16,17], food animals [18,19,20] and wild fauna [21] have been reported. Moreover, food may be contaminated [22,23].

The link between *C. difficile* and animals has been known for at least 60 years. In 1960, McBee [24] isolated this bacterium from the large intestine of a seal in Antarctica. By 1974 *C. difficile* had also been detected in animal faeces (donkeys, horses, cows and camels) and in the environment (hay, soil, sand and mud) [25]. In the early 1980s, *C. difficile* reservoirs were reported in healthy pigs and cattle [26,27] and in asymptomatic domestic pets, such as dogs, cats and birds, which had a prevalence of 21%, 30% and 33%, respectively [28]. Thus it was suggested that animals could be a vehicle of transmission to humans [29]. Interestingly, a common human pathogenic *C. difficile* ribotype (ribotype 078) was also isolated from pigs, cattle, and horses later, providing additional evidence of zoonotic transmission of *C. difficile* between animals and humans [30,31,32,33]. In more recent years, several studies have reported *C. difficile* in animals, on carcasses [21,34], in food processing facilities and in both raw and cooked foods [35,36,37,38,39,40,41].

Despite the increase in community-acquired CDI and data on *C. difficile* in the food chain, it is difficult to prove the source of infection in a given patient or outbreak as the same ribotypes and strains are common to both healthcare and food chain sources. Moreover, the patient may have acquired *C. difficile* sometime before the conditions in the colon changed to promote outgrowth. The objective of this review was to examine the evidence (CDI, virulence, ribotypes, environment, food animal and food sources and the current epidemiology of CDI in humans) supporting the hypothesis that *C. difficile* may be foodborne.

## 2. *C. difficile* Infection (CDI) in Humans

Elderly people are especially vulnerable to CDI, and cases are more likely to result in severe outcomes [42], possibly due to a decreased immune response or changes in the intestinal microbiota with age [43,44]. An underlying condition, chemotherapy or gastrointestinal surgery can increase susceptibility to CDI [45], which may become recurrent, leading to increased morbidity and mortality [46,47]. Broad-spectrum antibiotics significantly reduce the gut microflora diversity and alter the bile composition in the colon, facilitating CDI and recurrent infection in humans [48]. Treatment with acid suppression medication to prevent ulcers or treat acid-related diseases is also a risk factor for recurrence [49,50,51].

Metronidazole is used to treat mild to moderate CDI, while vancomycin is used in more severe cases, although the combination of both may be used when there are complications [52]. When these are ineffective, fidaxomicin has been proposed as an alternative to vancomycin [53,54] and has proven effective in preventing recurrent infection [55].

## 3. Virulence

**Figure 1:**
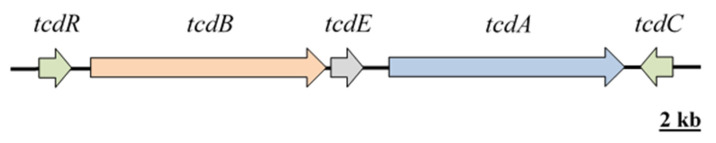
Illustration of the *C. difficile* Pathogenicity locus (PaLoc). Adapted from [65].

Within the host, *C. difficile* endospores germinate into vegetative cells, colonise the intestinal tract and produce toxins resulting in disease [56,57], which causes intestinal inflammation, perforation, toxic megacolon and pseudomembranous colitis [58,59]. Mortality rates range from less than 2% to 17% [60,61]. The main virulence factors in *C. difficile* are toxin A and toxin B, encoded by the *tcdA* (308 kDa) and *tcdB* (270 kDa) genes located on a pathogenicity locus (PaLoc) (Figure 1 and Table 1). Both are large clostridial glycosylation toxins and are activated in response to environmental signals during the late log and stationary phases. In addition to the toxins, two regulatory proteins (TcdR and TcdC) and a protein whose function remains unclear (TcdE) complete the PaLoc [62,63]. TcdR (also referred to as TcdD) is a positive regulator activated in stationary phase growth, while TcdC is a negative regulator produced during the exponential phase. Mutations, such as deletions in the *tcdC* gene, may cause increased production of toxins A and B [62,64].

TcdA and TcdB possess the same biological activities, among which is the disruption of the cytoskeleton that leads to cytopathic effects in cultured cells. They also possess proinflammatory activity and can stimulate intestinal epithelial cells and immune cells to produce cytokines and chemokines [66,67]. Even low doses of toxins A and B damage the tight junctions of the gut epithelial barrier, facilitating the translocation of commensal bacteria, inflammation and cell apotheosis [66,67,68]. Sequence variations, deletions, and duplications within the pathogenicity locus account for different toxinotypes of *C. difficile,* with 27 currently identified. Certain strains can present only one of the toxins genes (A^−^B^+^ or A^+^B^−^), however, they reportedly still cause severe disease in humans [62]. In addition, the cytotoxicitybetween toxins that belong to different toxinotypes may vary, making the relation between strain type and CDI severity even more complex [59]. Strains lacking toxin A are more frequently reported due to deletions in the receptor-binding repetitive regions of TcdA caused by the recombination between short repetitive sequences highly conserved in this toxin gene [63]. Donta et al. [66] reported TcdB to be 4 to 200-fold more cytotoxic than TcdA in a mouse model. Therefore, strains producing toxin B have a higher severity in humans.

Up to a third of *C. difficile* isolates also produce the transferase *C. difficile* binary toxin (CDT) [69,70]. CDT, composed of CDTa (biological activity) and CDTb (binding), inhibits the protein actin, damaging the cytoskeleton of the gastrointestinal tract (GIT) cells [71]. The presence of the full-length CDT locus implies the potential expression of the binary toxin, and although some strains contain portions of the CDT locus, these are predicted as non-binary toxin-producing strains [68,70]. CDT-producing strains have been previously associated with a higher production of toxins A and B, leading to an increased disease severity [71,72]. However, CDT is not always present in severe cases [73,74]. In addition, CDT can also be produced by only B^+^ and non-toxigenic strains (A^−^B^−^) [72]. Although CDT production is commonly associated with higher severity of *C. difficile* infection, the role of this toxin during infection and its mechanism of secretion is still not well understood.

**Table 1:** The virulence factors in *C. difficile* and their function.

Virulence Factor	Encoding Genes	Role in CDI	References
Toxin A	*tcdA*	Multiple cytopathic and cytotoxic effects on the targeted cells include disruption of Rho, Rac and Cdc42-dependent signalling, the actin cytoskeleton and the tight adherence junctions, increasing epithelial permeability, allowing commensal bacterial translocation, inflammation, diarrhoea and sometimes death.	[66,67,68,75]
Toxin B	*tcdB*		
TcdR	*tcdR*	TcdR is a positive regulator (produced in response environmental conditions) that triggers the induction of transcription of the toxin genes (*tcdA* and *tcdB*).	[76,77]
TcdC	*tcdC*	TcdC is a negative regulator that inhibits the expression of *tcdA* and *tcdB*. Mutations may cause increased production of toxins A and B.	[62,64]
TcdE	*tcdE*	TcdE may function as a lytic protein to facilitate the release of toxins A and B to the extracellular environment by a phage-like system, as these toxins lack signal peptides.	[78,79]
CDT	*cdtA & cdtB*	*C. difficile* binary toxin (CDT) is a transferase that disrupts the normal cytoskeletal function of cells by inhibiting the protein actin. The altered actin cytoskeleton causes an imbalance between actin and microtubules.	[69,70,71]

## 4. Ribotypes

There are in excess of 800 *C. difficile* ribotypes (RT), some of which are associated with increased virulence [6,80,81], including RT027 and RT078 [82,83]. These ribotypes are also more prevalent in human cases. RT027 (toxinotype III) has a mutation in *tcdC*, resulting in significantly increased production of toxins A and B while also carrying the genes encoding CDT production and fluoroquinolone resistance [84,85]. Although prevalence has decreased in Europe in recent years, RT027 is associated with a higher mortality and morbidity rate than other ribotypes [86]. The fluoroquinolone resistance, which emerged in two genetically distinct epidemiological lineages (FQR1 and FQR2), was a key driver in the rapid emergence of RT027 [57]. Moreover, this is essential to the increased severity of this ribotype, as this strain typically infects elderly hospital patients on fluoroquinolone treatment [5].

RT078 carries a 39 bp deletion in the *tcdC* gene and therefore overproduces toxins A and B in addition to the binary toxin CDT. In contrast to RT027, which is mostly hospital-acquired, RT078 is more prevalent in younger people and is generally associated with the community [87]. RT078 strains are resistant to fluoroquinolones and erythromycin, which has contributed to their higher prevalence in CDI [88]. Ribotype 126 has the same mutation in its *tcdC* gene found in RT078, is resistant to moxifloxacin and tetracycline and is also considered hypervirulent [89,90,91]. Other significant ribotypes from a public health perspective include RT017 and RT018. Although the former only produces toxin B, it is resistant to fluoroquinolones and rifampicin and has been associated with numerous outbreaks [92,93,94]. RT018 has high toxin production capacity, increased cell adhesion, is multidrug-resistant (erythromycin, clindamycin and moxifloxacin) and has become endemic in several countries, including Italy, Spain, Austria and Slovenia [95,96,97].

## 5. *C. difficile* in the Environment, Farm Animals and Food

### 5.1. Water

Toxigenic *C. difficile* has been isolated from a variety of aquatic environments, including drinking water, rivers, sewage effluent and swimming pools [98,99]. Coastal beaches and river sediments are also contaminated [98,99], in some cases by runoff from fields or effluents from wastewater treatment plants [100]. Indeed, *C. difficile* is often detected in water from treatment plants [101], and contamination of drinking water was the source of at least one *C. difficile* outbreak in Finland [102]. Thus, *C. difficile* survives in water and through the effluent treatment process [100].

### 5.2. Soil, Manure and Silage

*C. difficile* is commonly found in soil on farms as well as in forests, recreational parks, residential gardens, etc. [103,104,105,106,107]. These authors reported the highest prevalence in urban settings (57%), followed by farms (31%) and forests (28%). Shivaperumal et al. [108] found prevalence rates of 62%, 13% and 15% in garden soil, manure and compost, respectively, while Fröschle et al. [109] reported *C. difficile* to be the most prevalent *Clostridium* spp. in grass silage and cattle manure.

### 5.3. Farm Environment and Animals

Marcos et al. [110] reported that *C. difficile* were widespread in soil, water and faeces on beef, sheep and broiler farms, with the prevalence ranging from 7% to 83% and counts from 2.9 to 8.4 log_10_ cfu/g or /mL, depending on the animal species and sample type being tested. Other studies also found *C. difficile* in the faeces of a range of farm animals, including cattle, sheep, poultry and pigs [111,112,113,114,115,116]. Of these, pigs are the most important source of *C. difficile* [113,116], with the relative prevalence by age being 45%, 3% and 1% in suckling piglets, post-weaning piglets and finishing pigs, respectively [114]. Although these animals may show symptoms (diarrhoea), most are asymptomatic [114]. Other similar studies have reported a prevalence of 37% [115] and 78% [111] in piglets and 4% [115], 62% [117] and 9% [16] in mature pigs.

*C. difficile* are also found in cattle, especially younger animals. Rodriguez et al. [113] reported a prevalence of 11% in calves and 6% in adult cattle. Other studies have found these bacteria in 11% [118], 14% [117] and 22% of calves [111] and 7% of mature animals [16]. Sheep, including lambs, are also potential carriers, with 0.6 to 2% in the former and 7% reported in the latter [16,119].

Toxigenic *C. difficile* strains have also been reported in poultry faeces in several countries, including the USA (2.3%) [120], the Netherlands (5.8%) [107], Egypt (11.5%) [121], India (14%) [122], Zimbabwe (29%) [123] and Slovenia (62.3%) [124].

### 5.4. C. difficile at the Animal Slaughter Stage

Pathogenic bacteria in faeces on the hide/fleece or in the gastrointestinal tract are readily transferred to the carcass during slaughter and dressing [125]. *C. difficile* was found in 1%, 3% and 28% of porcine gut contents at slaughter in Belgium [126], Austria [18] and the Netherlands [127], respectively. Reported carcass contamination rates include 7% in Belgium [126], 15% in Canada [128] and 23% in Taiwan [129]. The prevalence of bovine carcass contamination ranges from 7–8% [111,126] but may be as high as 34% [130]. Ovine carcass contamination rates of 15% and 25% have been reported in Iran and Turkey, respectively [130,131]. While poultry carcass data is lacking, Candel-Pérez et al. [132] found *C. difficile* in 28% of gizzard and 6% of liver samples collected in a poultry processing plant in Spain. In Ireland, beef, sheep and broiler carcass contamination rates ranged from 40% to 100%, 40% to 60% and 10% to 40%, respectively, depending on the sampling stage during carcass processing [16].

Ribotypes 002, 005, 013, 014, 015, 019, 035, 062, 081, 087 and 126 have been identified in porcine faeces and rectal swabs at slaughter plants in Europe [18,111,126,127,133]. The *C. difficile* ribotypes isolated from other animal carcasses include 027 from cattle and IR46 from ovine carcasses [131]. Poultry slaughter data is lacking, although Koene et al. [16] found toxigenic ribotypes 056, 014 and 003 in faecal samples from poultry in Dutch slaughter plants.

### 5.5. C. difficile in Retail Foods

*C. difficile* has been reported in a range of foods at the retail stage. Thus, the consumption of contaminated retail foods, especially ready-to-eat (RTE) foods, is a risk factor for human infection [134]. Marcos et al. tested meat, dairy and vegetable retail foods and detected *C. difficile* in 9 out of the 240 samples tested [110]. These include corned beef (1), spinach leaves (2), iceberg and little gem lettuce (1 sample each), wild rocket, coleslaw, whole milk yoghurt and cottage cheese (also 1 sample each). Of these samples, direct counts were obtained for the spinach leaves (5.8 log_10_ cfu/g), coleslaw (4.3 log_10_ cfu/g) and cottage cheese (6.8 log_10_ cfu/g).

### 5.6. C. difficile in Meat and Seafood

Both raw and RTE meat and seafood are frequently contaminated with *C. difficile* [35,118], and the prevalence, including toxin gene profiles and ribotypes, is summarised in Table 2. The reported contamination rates include 41% [35] and 20% [135] for raw pork meat, 12% for ground pork meat [36] and up to 29% for pork sausages and RTE pork products [135]. A beef contamination rate of 42% was reported by Rodriguez-Palacios et al. [118], while ground beef rates include 2% [37], 12% [36], 20% [118] and 50% [35]. In one study, de Boer et al. [38] detected *C. difficile* in 6% of raw lamb samples. Reported poultry contamination rates include 1% [38], 3% [39], 8% [136,137], 13% [36,120] and 44% [35]. *C. difficile* has also been detected in shellfish and fish in several countries, with prevalence ranging from 4% to 49% [138,139,140,141].

### 5.7. C. difficile in Vegetables 

The information on *C. difficile* isolated from vegetables is summarised in Table 3, with overall prevalence rates of 2% to 5% [22,103,142]. Lim et al. detected *C. difficile* in 56% of organic and 50% of non-organic potatoes, 22% of organic beetroots, 56% of organic onions and 53% of organic carrots [143]. Tkalec et al. found this pathogen in 9% of leaf vegetables, 7% of ginger, 26% and 60% of potatoes, and 14.3% of homegrown leaf vegetables [144]. RTE salads contamination rates included 2% [41], 3% [142], 3.3% [145] and 8% (153].

All of these ribotypes have toxin genes associated with illness in humans. Many have been isolated directly from patients with CDI (Table 4), including 001, 002, 003, 010, 011, 012, 014, 015, 017, 018, 020, 023, 027, 029, 070, 071, 072, 077, 078, 087, 101, 126, 137 and 150. Of these, 002, 003, 012, 014, 027, 029, 070, 078 and 126 have been reported in confirmed community-acquired CDI, while 001, 017, 027, 072, 078 and 126 are hypervirulent.

### 5.8. Meta-Analysis 

**Figure 2:**
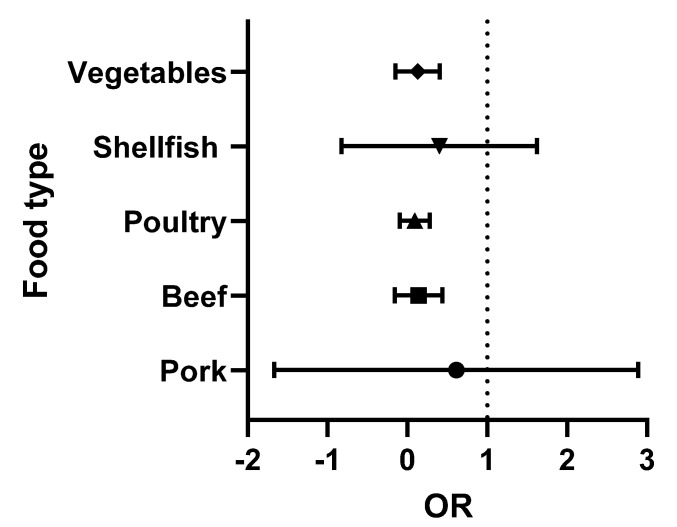
Forest plot of the OR of *C. difficile* (all ribotypes) in each food type.

**Figure 3:**
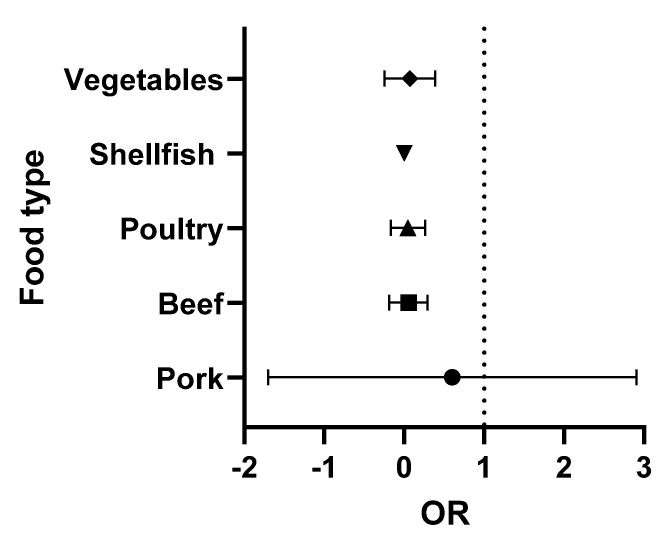
Forest plot of the OR of *C. difficile* 027 and 078 in each food type.

The data presented in Table 2 and Table 3 were analysed using Graphpad Prism version 9.3.1. The odds ratios (OR) (the odds of consuming a contaminated product) were calculated for each food type. Briefly, the OR was calculated as the number of positive over negative samples reported for each study. Turkey (with only two studies) was combined with the chicken data (poultry category), while the single lamb study was omitted. The medians and 95% confidence intervals were obtained and were then used to prepare the forest plots. In these Figures, the vertical line is set at an OR = 1 (50:50 chance of the food being contaminated). When all ribotypes are considered, shellfish and pork present a higher risk to the consumer (Figure 2). However, when the analysis is repeated, focusing exclusively on ribotypes 027 and 078 (the 2 hypervirulent strains most commonly associated with human infection), the increased risk is only associated with the consumption of pork (Figure 3).

## 6. The Epidemiology of Foodborne Infection

In 1978, *C. difficile* was recognised as the causative agent of pseudomembranous colitis and diarrhoea in patients on antimicrobial therapy and it was a hospital-associated disease [157]. In the 1980s and 1990s, the incidence of CDI increased significantly, driven by the use of broad-spectrum third-generation cephalosporins (to which *C. difficile* is intrinsically resistant), but the disease was rarely fatal [158,159]. There was a further increase in CDI in the first 10 years of this century driven by the emergence and epidemic spread of the hypervirulent strain, ribotype 027 [160]. The epidemiology of CDI also changed in terms of clinical presentation, response to treatment, and disease outcome. Community-acquired CDI, defined as cases with symptom onset in the community with no history of hospitalisation in the previous 12 weeks or symptom onset within 48 h of hospital admission [161], also emerged. Since then, the incidence of CDI has remained high in developed countries [159,162], and rates of community acquired CDI have increased, accounting for 41%, 30% and 14% of total CDI in the USA, Australia and the EU, respectively [96,159,163]. Furthermore, community acquired CDI patients are generally younger, healthy, often female and lack the traditional risk factors of CDI, including a history of antimicrobial usage [164].

The natural habitat of *C. difficile* is the mammalian gastrointestinal tract (GIT). These bacteria colonise the neonatal GIT, proliferate and are excreted in the faeces to which other newborn animals are exposed, and the cycle recommences. As mammals develop, other bacterial species colonise the GIT, and the prevalence of *C. difficile* decreases [165]. The GIT microbiota inhibit germination, vegetative growth and toxin production, thus protecting against *C. difficile* [48]. However, in the 1990s, this protection was removed when cephalosporins were used in animal husbandry, and food animals became a major reservoir and amplification host for *C. difficile* [119,166], resulting in the contamination of the environment and a range of foods [100,119,166].

**Figure 4:**
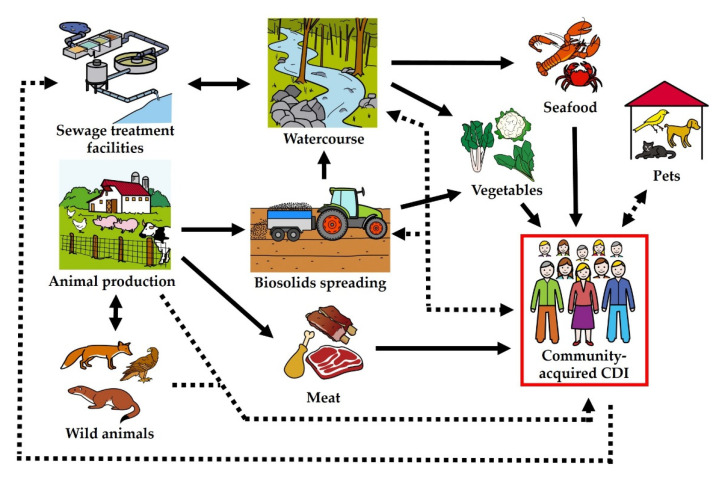
The cycle of community-associated CDI infections from zoonotic, environmental or foodborne sources. Adapted from [100] using ARASAAC pictograms.

Once the environment is contaminated, there are multiple direct and indirect routes to humans, including via food (as illustrated in Figure 4). It is all but impossible to provide incontrovertible proof of foodborne transmission because of the ubiquitous nature of *C. difficile*, delayed onset of symptoms, ability to persist for extended periods as an endospore, etc. However, it has been shown that *C. difficile* endospores in animal waste, wastewater treatment sludge, soil, manure and compost may survive for extended periods of time, facilitating direct contamination of vegetables and fruit or meat via cross-contamination of carcasses during slaughter and processing [108,147]. Water also frequently contains *C. difficile* endospores [99,100,148], and food production may also be contaminated via water used for irrigation or food processing [100,144]. Moreover, the presence of endospores in rivers may contaminate fish and seafood [100,138,139,141]. Transfer from food and wild animals and from domestic pets has also been described [116,149].

Of particular interest, from the public health perspective, is the detection of similar *C. difficile* isolates in farm animals and in humans suffering from CDI, suggesting this pathogen may be zoonotic [150]. Whole genome sequencing (WGS) analysis has shown that ribotypes 078, 126 and 066, commonly found in pigs and/or cattle, are genetically identical to those in humans [151,152,153,154,155]. Although ribotype data for sheep is limited, ribotypes 014, 010 and 045 are common to both humans and ovine sources [119,156,167], while human-related ribotypes 001, 014 and 039 are also found in broilers [120,121,122,168].

## 7. Control Strategies

CDI can be controlled in hospitals using deep environmental cleaning, appropriate hand hygiene, stringent infection control and antimicrobial stewardship [169]. However, the same strategies cannot be used in agriculture and food processing [15]. Reduced usage of antibiotics in food animal production would reduce *C. difficile* amplification but is unlikely as increasing global food demand is driving increased antimicrobial usage in animal husbandry, which is projected to rise by 67% by 2030 [15,140]. In 2006 the EU banned the use of antibiotics as growth promoters, followed by the USA in 2017, but other major food-producing countries still allow this practice [170].

Preventing the recycling and dissemination of *C. difficile* endospores in animal slurries applied to land as organic fertilisers would also facilitate reduced environmental contamination and animal carriage. However, research is required to develop effective treatments [171]. Vaccination of food-producing animals is another possible control strategy, but an effective vaccine has not been developed yet [15]. Controlling *C. difficile* in food is dependent on reducing or eliminating the endospores, which are resistant to chilled (4 °C) and freezing (−18 °C and −80 °C) temperatures [172,173]. Although the endospores are resistant at 80 °C [172,173,174] and will survive the recommended cooking time temperature combinations recommended for meat [174], they are eliminated at 98 °C for 2 min [175]. The same authors suggested microwave irradiation (800 W/60 s) also achieved complete inactivation by denaturing the outer coat.

*C. difficile* endospores are also resistant to desiccation, hydrostatic pressure [37,176,177,178,179] and a range of food preservatives, including sodium nitrite, sodium nitrate and sodium metabisulfite, at permitted concentrations [180]. In contrast, nisin [181], black seed oil, myrrh water [182], garlic juice, peppermint oil, trans-cinnamaldehyde, allicin, menthol and zingerone [183] have a potential application, but validation studies are required before they can be used in controlling *C. difficile* in food.

## 8. Conclusions

Based on the information provided, it was concluded that *C. difficile* is widespread in the environment and along the food chain. Many food isolates carry the virulence factors required for human infection, and there is no conceivable reason why food is not a source of these pathogens. This conclusion is further supported by the presence of the same ribotypes in food and humans suffering from community-acquired CDI. Based on our analysis, potentially vulnerable consumers should be advised not to handle or consume shellfish or pork.

## Figures and Tables

**Table 2:** Meat and seafood retail foods contaminated with *C. difficile*, including toxin gene profiles (toxins A, B and CDT) and ribotypes.

Product	Raw or RTE	Total No. (%) Positive	Toxin Gene Profile	Ribotype(s)	Reference
Ground pork	Raw	3/7 (41.3%)	A^+^ B^+^ CDT^+^	027 078	[35]
Ground pork	Raw	14/115 (12%)	A^+^ B^+^ CDT^+^	027 078	[36]
Ground pork	Raw	2/66 (3.0%)	A^+^ B^+^ CDT^−^	029	[39]
Pork meat	Raw	35/303 (11.5%)	A^+^ B^+^ CDT^+^	078	[136]
Pork sausages	RTE	10/16 (62.5%)	A^+^ B^+^ CDT^+^	027 078	[35]
Ground beef	Raw	13/26 (42.4%)	A^+^ B^+^ CDT^+^	027 078	[35]
Ground beef	Raw	11/53 (20.8%)	A^+^ B^+^ CDT^+^	M31	[118]
			A^+^ B^+^ CDT^−^	014 077	
Ground beef	Raw	14/115 (12%)	A^+^ B^+^ CDT^+^	027 078	[36]
Ground beef	Raw	2/105 (1.9%)	A^+^ B^+^ CDT^ND^	012	[37]
Ground beef	Raw	21/303 (6.9%)	A^+^ B^+^ CDT^+^	PA22	[136]
Beef	Raw	1/67 (1.5%)	A^+^ B^+^ CDT^−^	029	[39]
Beef sausages	RTE	1/7 (14.3%)	A^+^ B^+^ CDT^+^	027	[35]
Corned beef	RTE	1/10 (10%)	A^ND^ B^+^ CDT^ND^	ND ^1^	[110]
Ground veal	Raw	1/7 (14.3%)	A^+^ B^+^ CDT^+^	M31	[118]
Turkey	Raw	44/303 (14.5%)	A^+^ B^+^ CDT^+^	PA01 PA05 PA16	[136]
Ground turkey	Raw	4/9 (44.4%)	A^+^ B^+^ CDT^+^	078	[35]
Lamb	Raw	1/16 (6.3%)	A^+^ B^+^ CDT^+^	045	[38]
Chicken	Raw	7/257 (2.7%)	A^+^ B^+^ CDT^−^	001 003 071 087	[38]
Chicken	Raw	1/67 (1.5%)	A^+^ B^+^ CDT^−^	029	[39]
Chicken	Raw	25/310 (8.0%)	A^+^ B^+^ CDT^−^	ND ^1^	[137]
Chicken	Raw	26/203 (12.8%)	A^+^ B^+^ CDT^+^	078	[23]
Chicken	Raw	24/303 (7.8%)	A^+^ B^+^ CDT^+^	PA05 PA16	[136]
Chicken	Raw	4/32 (12.5%)	A^+^ B^+^ CDT^+^	078	[110]
Chicken	RTE	1/130 (0.8%)	A^+^ B^+^ CDT^−^	014 020	[41]
Shellfish	Raw	118/702 (16.8%)	A^+^ B^+^ CDT^+^	126 475	[141]
Bivalve molluscs	Raw	26/53 (49%)	A^+^ B^+^ CDT^+^	078	[139]
			A^+^ B^+^ CDT^−^	002 012 014/020 018 001	
Bivalve molluscs	Raw	36/925 (3.9%)	A^+^ B^+^ CDT^+^	078 126 010	[140]
			A^−^ B^+^ CDT^−^	017 001	

+: Positive; −: Negative; ^1^ ND: Not determined.

**Table 3:** Vegetable retail foods contaminated with *C. difficile,* including toxin gene profiles (toxins A, B and CDT) and ribotypes.

Product	Raw or RTE	Total No. (%) Positive	Toxin Gene Profile	Ribotype(s)	Reference
Root vegetables (potatoes, beetroots, onions and carrots)	Raw	30/100 (30%)	A^+^ B^+^ CDT^+^	QX 274	[143]
			A^+^ B^+^ CDT^−^	002 137 QX519 QX049 101	
			A^−^ B^+^ CDT^+^	070 237 584	
			A^−^ B^−^ CDT^+^	033	
Root vegetables (potatoes, ginger) and leaf vegetables	Raw and RTE	28/154 (18.2%)	A^+^ B^+^ CDT^−^	001/072 011/049 014/020 012 070 150 394 SLO129 SLO187 SLO279	[144]
			A^+^B^+^CDT^+^	027 244 126 023	
Lettuce	RTE	1/54 (1.9%)	A^+^ B^+^ CDT^+^	126	[41]
Vegetables (potato, onion, mushroom, carrot, radish and cucumber)	Raw	7/300 (2.4%)	A^+^ B^ND^ CDT ^ND^	ND ^1^	[103]
Salad (lettuce, lamb’s lettuce) and vegetable (pea sprouts)	RTE	3/104 (2.8%)	A^+^ B^+^ CDT^−^	014/020 001 015	[142]
Vegetables (carrots, potatoes, garlic, ginger, beets, mushrooms, lettuce, green onions, radishes, etc.)	Raw and RTE	5/111 (4.5%)	A^+^ B^+^ CDT^+^	078	[22]
			A^+^ B^+^ CDT^−^	ND ^1^	
Salad (baby leaf spinach)	RTE	2/60 (3.3%)	A^+^ B^+^ CDT^+^	078 126	[145]
Salad (baby leaf spinach, organic mixed leaf salad, organic lettuce)	RTE	3/40 (7.5%)	A^+^ B^+^ CDT ^ND^	001	[146]
			A^−^ B^+^ CDT ^ND^	017	
Spinach leaves	RTE	2/10 (20%)	A^−^B^+^ CDT^−^	ND ^1^	[110]
Iceberg lettuce leaves	RTE	1/10 (10%)	A^−^B^+^ CDT^−^	ND ^1^	[110]
Little Gem lettuce leaves	RTE	1/10 (10%)	A^−^B^+^ CDT^−^	ND ^1^	[110]
Wild rocket leaves	RTE	1/10 (10%)	A^−^B^+^ CDT^+^	ND ^1^	[110]
Coleslaw	RTE	1/10 (10%)	A^−^B^+^ CDT^−^	ND ^1^	[110]

+: Positive; −: Negative; ^1^ ND: Not determined.

**Table 4:** Further characterisation (pathogenicity, hypervirulence and association with community-acquired CDI) of the ribotypes isolated from foods (Table 2 and Table 3).

Ribotype	Pathogenic			Hypervirulent			CA CDI ^1^	Reference(s)
	yes	no	unk ^2^	yes	no	unk		
001	✓			✓				[81,99,147,148,149,150]
002	✓				✓		✓	[81,99,148,149,151,152]
003	✓				✓		✓	[81,99]
010	✓				✓			[150]
011	✓				✓			[148]
012	✓				✓		✓	[81,148,149,150,153]
014	✓				✓		✓	[81,99,148,151,153]
015	✓				✓			[148,149,151]
017	✓			✓				[145,148,149,154]
018	✓				✓			[148,149]
020	✓				✓			[148,149,151]
023	✓				✓			[148,149,151]
027	✓			✓			✓	[72,73,147,148,149,153,155]
029	✓				✓		✓	[99,153]
070	✓				✓		✓	[81,149]
071	✓				✓			[149]
072	✓			✓				[99,148,149,156]
077	✓				✓			[149]
078	✓			✓			✓	[72,73,148,149,153,154,155]
087	✓			✓				[148,149]
101	✓				✓			[149]
126	✓			✓			✓	[6,80,81,99,149,153]
137	✓				✓			[149]
150	✓				✓			[149]
033, 045, 049, 237, 244, 394, 475, 584, M31, PA01, PA05, PA16, PA22, QX049, QX274, QX519, SLO129, SLO187, SLO279						No information		

^1^ CA CDI = community acquired *C. difficile* infection; ^2^ unk = unknown.

## Data Availability

Not applicable.
